# The infraacetabular screw versus the antegrade posterior column screw in acetabulum fractures with posterior column involvement: a biomechanical comparison

**DOI:** 10.1007/s00402-024-05324-3

**Published:** 2024-04-27

**Authors:** Nico Hinz, Dirk Baumeister, Julius Dehoust, Matthias Münch, Karl-Heinz Frosch, Peter Augat, Maximilian J. Hartel

**Affiliations:** 1 Department of Trauma and Orthopaedic Surgery, University Medical Center Hamburg-Eppendorf, Martinistrasse 52, 20246 Hamburg, Germany; 2 Department of Trauma Surgery, Orthopaedics and Sports Traumatology, BG Klinikum Hamburg, Bergedorfer Strasse 10, 21033 Hamburg, Germany; 3 Institute for Biomechanics, BG Unfallklinik Murnau, Professor-Kuentscher-Strasse 8, 82418 Murnau am Staffelsee, Germany; 4 Laboratory for Biomechanics, BG Klinikum Hamburg, Bergedorfer Strasse 10, 21033 Hamburg, Germany

**Keywords:** Acetabulum fracture, Anterior column plus posterior hemitransverse fracture, Antegrade posterior column screw, Infraacetabular screw, Suprapectineal plate, Biomechanical testing

## Abstract

**Introduction:**

Traditionally, plate osteosynthesis of the anterior column combined with an antegrade posterior column screw is used for fixation of anterior column plus posterior hemitransverse (ACPHT) acetabulum fractures. Replacing the posterior column screw with an infraacetabular screw could improve the straightforwardness of acetabulum surgery, as it can be inserted using less invasive approaches, such as the AIP/Stoppa approach, which is a well-established standard approach. However, the biomechanical stability of a plate osteosynthesis combined with an infraacetabular screw instead of an antegrade posterior column screw is unknown.

**Material and methods:**

Two osteosynthesis constructs were compared in a synthetic hemipelvis model with an ACPHT fracture: Suprapectineal plate + antegrade posterior column screw (APCS group) vs. suprapectineal plate + infraacetabular screw (IAS group). A single-leg stance test protocol with an additional passive muscle force and a cyclic loading of 32,000 cycles with a maximum effective load of 2400 N was applied. Interfragmentary motion and rotation of the three main fracture lines were measured.

**Results:**

At the posterior hemitransverse fracture line, interfragmentary motion perpendicular to the fracture line (*p* < 0.001) and shear motion (*p* < 0.001) and at the high anterior column fracture line, interfragmentary motion longitudinal to the fracture line (*p* = 0.017) were significantly higher in the IAS group than in the APCS group. On the other hand, interfragmentary motion perpendicular (*p* = 0.004), longitudinal (*p* < 0.001) and horizontal to the fracture line (*p* = 0.004) and shear motion (*p* < 0.001) were significantly increased at the low anterior column fracture line in the APCS group compared to the IAS group.

**Conclusions:**

Replacing the antegrade posterior column screw with an infraacetabular screw is not recommendable as it results in an increased interfragmentary motion, especially at the posterior hemitransverse component of an ACPHT fracture.

## Introduction

The incidence of acetabulum fractures in Germany raised from 2009 to 2019 by + 58% due to an aging society with a higher risk of fragility- and osteoporosis-associated fractures [1, 2]. Especially in elderly patients, the main trauma mechanism for acetabulum fractures is a ground-level fall on the greater trochanter resulting in an anterior column type fracture frequently associated with a posterior hemitransverse fracture line, a so-called anterior column plus posterior hemitransverse (ACPHT) fracture [2–7].

The incidence of a posttraumatic osteoarthritis after surgically treated acetabulum fractures is up to 30% [8–10]. An exact anatomical reduction and a biomechanically stable osteosynthesis are crucial for the survival of the hip joint, since they are the main predictors for clinical and radiological outcome of acetabulum fractures [8, 11–13]. Especially in geriatric patients with osteoporotic bone, a biomechanically stable osteosynthesis was found to be important for the radiological outcome [7]. Additionally in elderly patients, a biomechanically stable fracture fixation is important to ensure early mobilization to prevent immobilization-associated complications and mortality [14–16].

The surgical treatment of ACPHT fractures usually requires open reduction and internal stabilization of both columns [17]. Traditionally, fixation of the anterior column is performed by plate and screw osteosyntheses and fixation of the posterior column by an antegrade posterior column screw via an ilioinguinal approach as a standard [18–24]. In the last few years, a trend towards less invasive techniques in acetabular fracture surgery could be observed [25–28]. The use of less invasive approaches has been proven to be beneficial, especially in geriatric patients, which is why the ilioinguinal approach has been replaced by the modified Stoppa (AIP) approach as well as the Pararectus approach [18, 23, 25, 28–32]. However, the implantation of an antegrade posterior column screw requires an additional small incision to the first ilioinguinal window, if the plate osteosynthesis is performed via the modified Stoppa approach, while this will be not necessary with the Pararectus approach [19, 21, 31, 33, 34].

The so-called infraacetabular screw was first described by Culeman et al. in 2011 as a transfixation of both columns through an additional pathway anterior and inferior to the acetabulum [35]. Several biomechanical studies demonstrated, that an infraacetabular screw augments the biomechanical stability of osteosynthetically stabilized fractures involving both columns [36–39]. The infraacetabular screw can be inserted using all established less invasive approaches [19, 34, 40]. Consequently, if the antegrade posterior column screw could be replaced by an infraacetabular screw for stabilization of ACPHT fractures, the surgical straightforwardness would further be improved. However, the biomechanical stability of a plate osteosynthesis of the anterior column combined with an infraacetabular screw instead of an antegrade posterior column screw is unknown so far. Hence, the aim of this biomechanical study is to compare a plate osteosynthesis of the anterior column plus an antegrade posterior column screw with a plate osteosynthesis of the anterior column plus an infraacetabular screw in a synthetic hemipelvis model of an ACPHT fracture. We hypothesized that the infraacetabular screw has an inferior biomechanical stability compared to the antegrade posterior column screw.

## Materials and methods

### Fracture model and osteosynthesis constructs

In this study, 12 synthetic left hemipelves (Composite, 17 PCF solid foam core, 4th generation, Sawbones, Malmö, Sweden) were used. An anterior column plus posterior hemitransverse fracture was created in a standardized manner with an oscillating saw as described previously by Tanoglu et al. [41]. Afterwards, the 12 hemipelves were randomly assigned to one of the following groups:

1. APCS group (n = 6): Suprapectineal plate osteosynthesis plus an antegrade posterior column screw
2. IAS group (n = 6): Suprapectineal plate osteosynthesis plus an infraacetabular screw

**Fig. 1:**
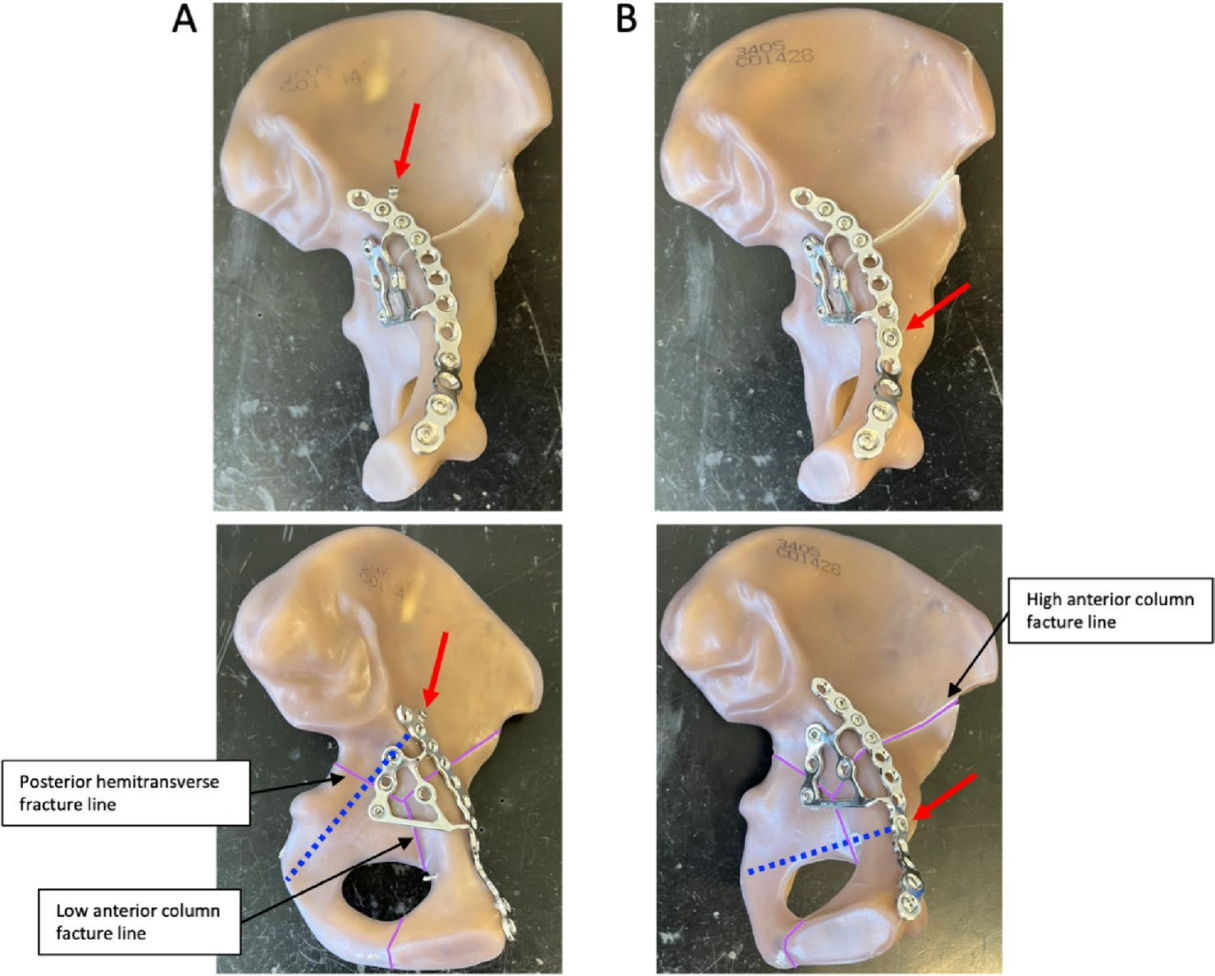
Fixation of a standardized generated anterior column plus posterior hemitransverse fracture with a suprapectineal plate plus an antegrade posterior column screw (**A**) or plus an infraacetabular screw (**B**). Red arrows indicate either the antegrade posterior column screw or the infraacetabular screw and the blue doted lines their respective course. The fracture lines are outlined in purple and the three main fracture lines are labeled

In both groups, the fracture was stabilized with a suprapectineal plate with quadrilateral buttress (PRO Pelvis and Acetabulum System, Stryker, Amsterdam, Netherlands) as the basic osteosynthesis. Bicortical 3.5 mm screws were used for fixation of the plate as it is shown in Fig. 1. For the APCS group, a 3.5 mm antegrade posterior column screw was inserted as described previously by Jung et al. outside the plate [21]. For the IAS group, a 3.5 mm infraacetabular screw was inserted as described previously by Baumann et al. through the fifth plate hole from ventral [40]. Both osteosynthesis constructs are illustrated in Fig. 1.

### Test setup

Biomechanical testing was performed with a single-leg stance model as described by Becker et al. [42]. As recently described in a review, the biomechanical test setup of Becker et al. is an optimal model to simulate the physiological forces and force angles of the hip joint, since it is based on the observations made by Bergmann et al. in vivo with telemetric hip implants [43–45].

**Fig. 2:**
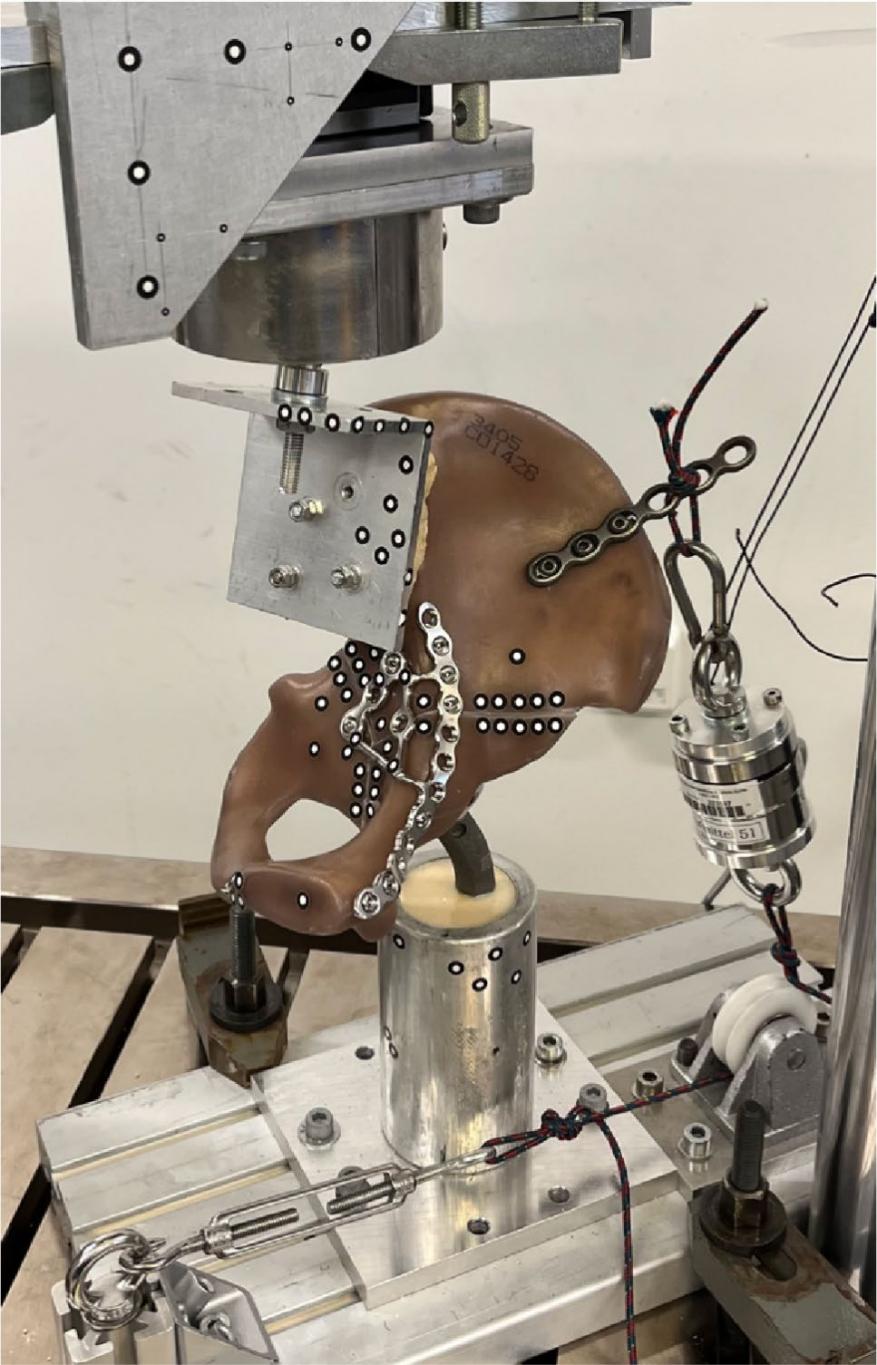
Biomechanical test setup. The hemipelvis was mounted proximally via a sacrum substitute with a ball joint to an electro-dynamic test machine. Distally the hemipelvis was connected to a prosthesis stem with a femoral head. The hemipelvis, which was thus freely movable in all three axes, was stabilized by an additional tension mechanism via a plate at the os ilium

The hemipelvis was rigidly connected to a sacrum substitute (polyurethane cast, RenCast FC 53 A/B, Gößl + Pfaff GmbH, Karlskron/Brautlach, Germany) via three threaded rods with corresponding nuts to ensure an equal load distribution between the testing machine and the synthetic hemipelvis. The sacrum substitute was connected via a ball joint, which consisted of a 36 mm diameter ceramic ball and an artificial acetabular cup embedded in an aluminum cylinder, to a linear slide. The linear slide in turn was attached to the actuator of an electro-dynamic testing machine (LTM10 T, ZwickRoell AG, Ulm, Germany). Force was applied proximally via the testing machine to the hemipelvis. The linear slide ensured a vertical force direction by enabling lateral movements, and in combination with the ball joint, movement of the hemipelvis in all three axes was allowed. Distally the hemipelvis was moveably connected via a 28 mm ball and a 55 mm dual head cup to a hip prosthesis revision stem, which was embedded in an aluminum cylinder. An additional tension mechanism with a 3 mm UHMWPE fiber cord (LIROS GmbH, Berg, Germany) was mounted via a plate and three screws at the ala of the os ilium to prevent collapsing of the hemipelvis and to generate a secondary, passive force mimicking the main muscle forces at the pelvis. A load cell (U3, measuring range 1 kN, HBM GmbH, Darmstadt, Germany) was used to measure the additional, secondary force and a turnbuckle was used to adapt the length of the tension mechanism to achieve physiological loading angles, as observed by Bergman et al. [43–45]. The effective loading force at the acetabulum was the sum of the axially applied primary loading via the electro-dynamic testing machine and of the secondary, passive load resulting from the tension mechanism. Figure 2 illustrates the biomechanical test setup.

### Testing protocol

A cyclic sinusoidal fatigue test protocol with periodically increasing loading forces was applied to the synthetic hemipelves as described by Becker et al. [42]. The lower level set point of the axially applied sinusoidal cyclic loading via the electro-dynamic testing machine was set to 50 N and the initial upper level set point was 250 N. The upper level set point was increased by 50 N every 1000 cycles up to a maximum of 1800 N. This resulted in a total of 32,000 cycles. The loading frequency was 1 Hz. The secondary, passive load ranged between 500 and 600 N when the final primary load of 1800 N were applied resulting in an effective load at the acetabulum of 2300–2400 N. These loading forces are comparable to the maximum resulting hip loads measured by Bergmann et al. with telemetric hip implants during normal walking [43–45]. A sudden loss of resistance and/or an (out-)breakage of the osteosynthesis construct were defined as stop criteria for the biomechanical testing.

### Measurement system and measurement variables

**Fig. 3:**
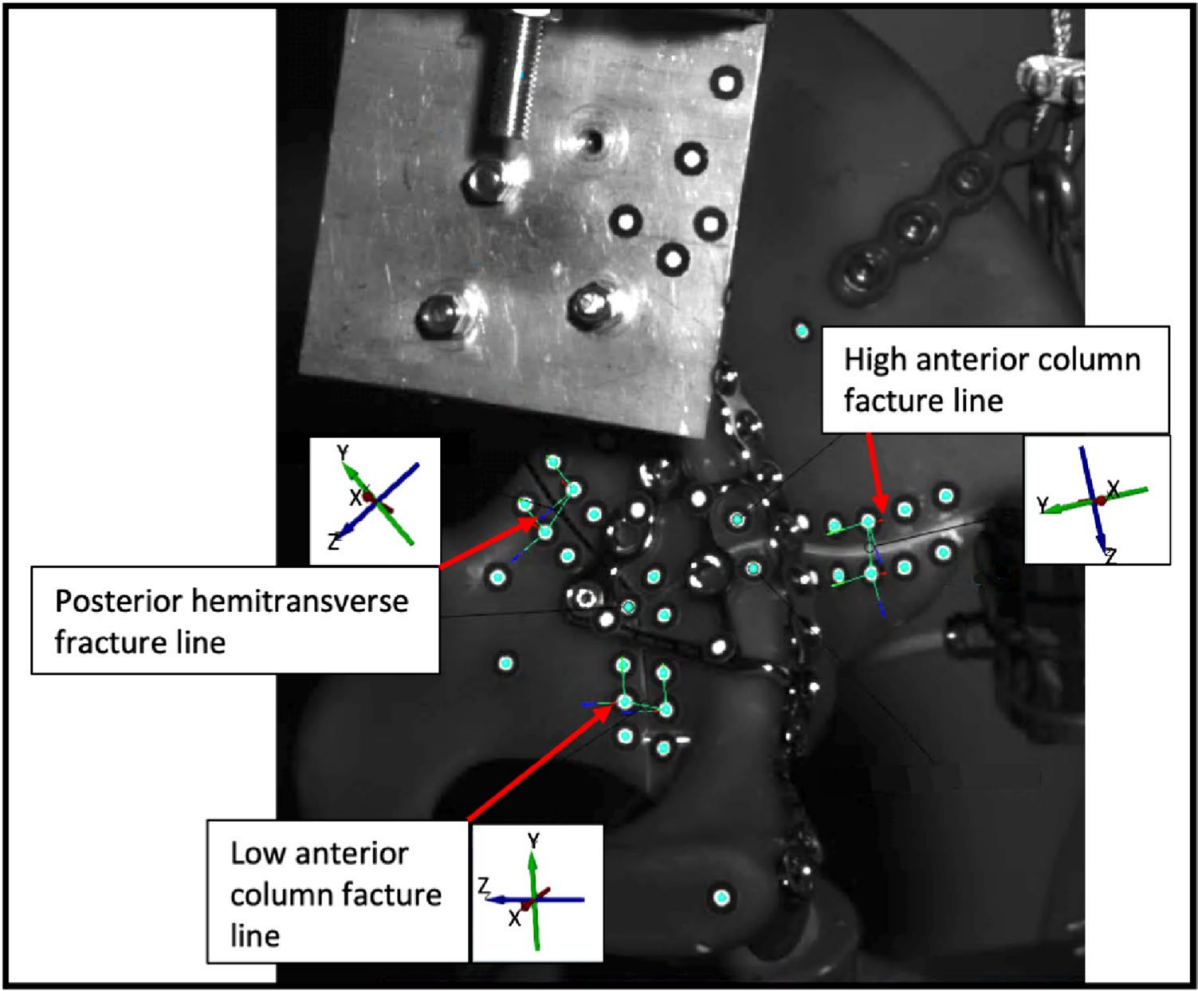
Analysis of interfragmentary motion and interfragmentary rotation of the three main fracture lines (posterior hemitransverse fracture line, high anterior column fracture line, low anterior column fracture line) with a 6-DoF measurement with regard to the corresponding coordinate systems shown

Measurements were performed with a 3D motion analysis video system (ARAMIS 5 M, Carl Zeiss GOM Metrology GmbH, Braunschweig, Germany) with tracked passive optical markers along the three main fracture lines: Posterior hemitransverse fracture line, high anterior column fracture line and low anterior column fracture line (Fig. 3). The GOM Correlate Pro software was used to analyze interfragmentary motion and interfragmentary rotation. Therefore, a six degrees of freedom (6-DoF) analysis was performed for each of the three fracture lines using the local coordinate systems and rigid-body motion correction. This results in three measurement variables for interfragmentary motion (LX [mm], LY [mm] and LZ [mm]) and three measurement variables for interfragmentary rotation (Phi(X) [°], Theta(Y) [°], Psi(Z) [°]) along each of the three axes. Additionally, shearXY [mm] was calculated according to the following formula:

$$\sqrt {LX^2 + LY^2 }$$

Figure 3 illustrates the three main fracture lines and their corresponding coordinate systems, on the basis of which interfragmentary motion and rotation were assessed.

### Statistical analysis

Statistical analysis and data visualization were performed using GraphPad Prism 9 (GraphPad Software, La Jolla, CA, USA). Interfragmentary motion and rotation were compared between the APCS and the IAS group and were calculated as the difference between the 32,000th cycle with a primary load of 1800 N and the initial primary loading with 50 N at the start of cyclic loading protocol. Hence, the interfragmentary motion reported in this study represents a combined elastic–plastic deformation. Unpaired, two-tailed Student’s t-test was used for comparison. Data are presented as mean ± standard deviation unless otherwise stated. Exact *p*-values are reported unless *p* < 0.001 and *p*-values of ≤ 0.05 were considered statistically significant.

## Results

A stop criterion of the biomechanical testing (sudden loss of resistance and/or (out-)breakage of the osteosynthesis construct) was not reached in any of the 12 hemipelves. Table 1 shows the comparison of interfragmentary motion and rotation for the three main fracture lines between the APCS group and the IAS group at a primary load with 1800 N after the 32,000th cycle in relation to the initial primary loading with 50 N at the start of cyclic loading protocol.

**Table 1:** Comparison of interfragmentary motion and interfragmentary rotation along the three axes between the APCS group and the IAS group for the three main fracture lines

	Antegrade posterior column screw (n = 6)	Infraacetabular screw (n = 6)	*p*-value
Posterior hemitransverse fracture line			
Interfragmentary motion			
Shear XY [mm]	0.305 ± 0.173	1.174 ± 0.356	** < 0.001**
LX [mm]	0.284 ± 0.198	1.156 ± 0.337	** < 0.001**
LY [mm]	0.049 ± 0.050	0.172 ± 0.169	0.119
LZ [mm]	0.121 ± 0.075	0.111 ± 0.096	0.840
Interfragmentary rotation			
Phi(X) [°]	0.376 ± 0.241	0.244 ± 0.190	0.319
Theta(Y) [°]	0.381 ± 0.352	0.547 ± 0.486	0.513
Psi(Z) [°]	0.522 ± 0.486	0.643 ± 0.347	0.631
High anterior column fracture line			
Interfragmentary motion			
Shear XY [mm]	0.814 ± 0.205	0.966 ± 0.599	0.571
LX [mm]	0.800 ± 0.218	0.875 ± 0.639	0.792
LY [mm]	0.116 ± 0.076	0.324 ± 0.161	**0.017**
LZ [mm]	0.574 ± 0.339	0.708 ± 0.431	0.562
Interfragmentary rotation			
Phi(X) [°]	0.315 ± 0.079	0.488 ± 0.279	0.174
Theta(Y) [°]	0.390 ± 0.316	0.293 ± 0.176	0.524
Psi(Z) [°]	0.267 ± 0.147	0.621 ± 0.408	0.074
Low anterior column fracture line			
Interfragmentary motion			
Shear XY [mm]	0.612 ± 0.152	0.183 ± 0.125	** < 0.001**
LX [mm]	0.455 ± 0.130	0.177 ± 0.126	**0.004**
LY [mm]	0.408 ± 0.083	0.032 ± 0.037	** < 0.001**
LZ [mm]	0.183 ± 0.060	0.072 ± 0.039	**0.004**
Interfragmentary rotation			
Phi(X) [°]	0.271 ± 0.265	0.234 ± 0.199	0.789
Theta(Y) [°]	0.684 ± 0.394	0.474 ± 0.343	0.348
Psi(Z) [°]	0.306 ± 0.075	0.364 ± 0.349	0.696

Data are presented as mean ± standard deviation. A *p*-value ≤ 0.05 is considered statistically significant. *p*-values indicating a statistically significant difference are highlighted in bold

For the posterior hemitransverse fracture line, interfragmentary motion along the X-axis (LX [mm]; APCS: 0.284 ± 0.198 vs. IAS: 1.156 ± 0.337; *p* < 0.001) and shear in XY direction (shearXY [mm]; APCS: 0.305 ± 0.173 vs. IAS: 1.174 ± 0.356; *p* < 0.001) were significantly higher in the IAS group compared to the APCS group. All other variables for interfragmentary motion and interfragmentary rotation did not show a significant difference between both groups regarding the posterior hemitransverse fracture line.

Interfragmentary motion and interfragmentary rotation at the high anterior column fracture line were also not significantly different between both groups, except of a significantly elevated interfragmentary motion along the Y-axis in the IAS group compared to the APCS group (LY [mm]; APCS: 0.116 ± 0.076 vs. IAS: 0.324 ± 0.161; *p* = 0.017).

**Fig. 4:**
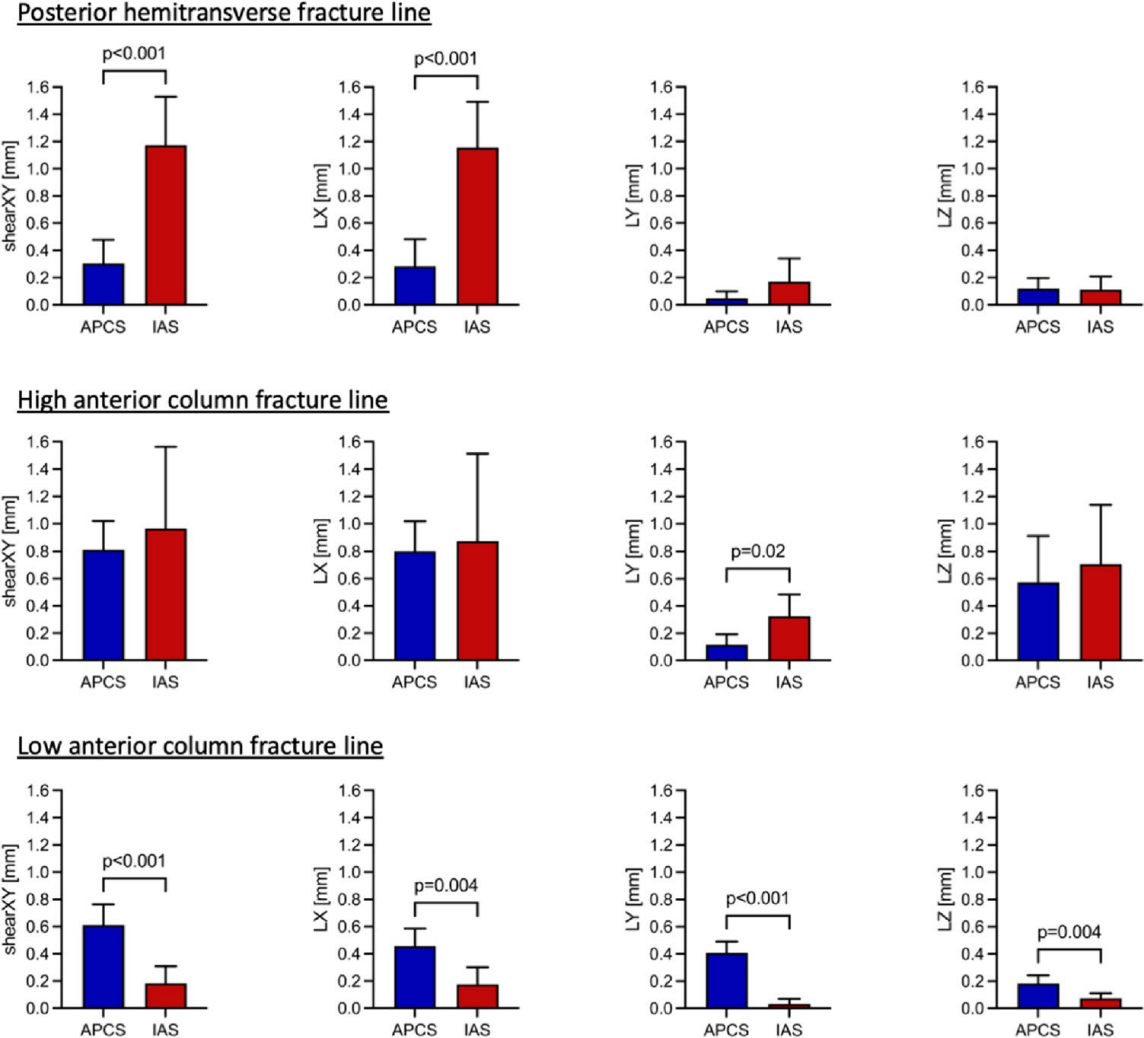
Interfragmentary motion along the three axes of the antegrade posterior column screw group and the infraacetabular screw group for the three main fracture lines. Columns represent mean and error bars indicate standard deviation. *p*-values ≤ 0.05 are considered statistically significant

For the low anterior column fracture line, interfragmentary motion along the X-axis (LX [mm]; APCS: 0.455 ± 0.130 vs. IAS: 0.177 ± 0.126; *p* = 0.004), Y-axis (LY [mm]; APCS: 0.408 ± 0.083 vs. IAS: 0.032 ± 0.037; *p* < 0.001) and Z-axis (LZ [mm]; APCS: 0.183 ± 0.060 vs. IAS: 0.072 ± 0.039; *p* = 0.004) as well as shear in XY direction (shearXY [mm]; APCS: 0.612 ± 0.152 vs. IAS: 0.183 ± 0.125; *p* < 0.001) were significantly higher in the APCS group compared to the IAS group. Interfragmentary rotation at the low anterior column fracture line was not significantly different between both groups. Figure 4 illustrates interfragmentary motion of the APCS group and IAS group for the three main fracture lines.

## Discussion

This biomechanical study revealed that an antegrade posterior column screw provides a superior biomechanical stability compared to an infraacetabular screw when focusing on the posterior column in acetabulum fractures. Therefore, the hypothesis that an infraacetabular screw has an inferior biomechanical stability compared to the antegrade posterior column screw in fixation of acetabulum fractures with posterior column involvement was confirmed. Consequently, the infraacetabular screw should not be mistaken as a possible replacement for the traditional antegrade posterior column screw.

**Fig. 5:**
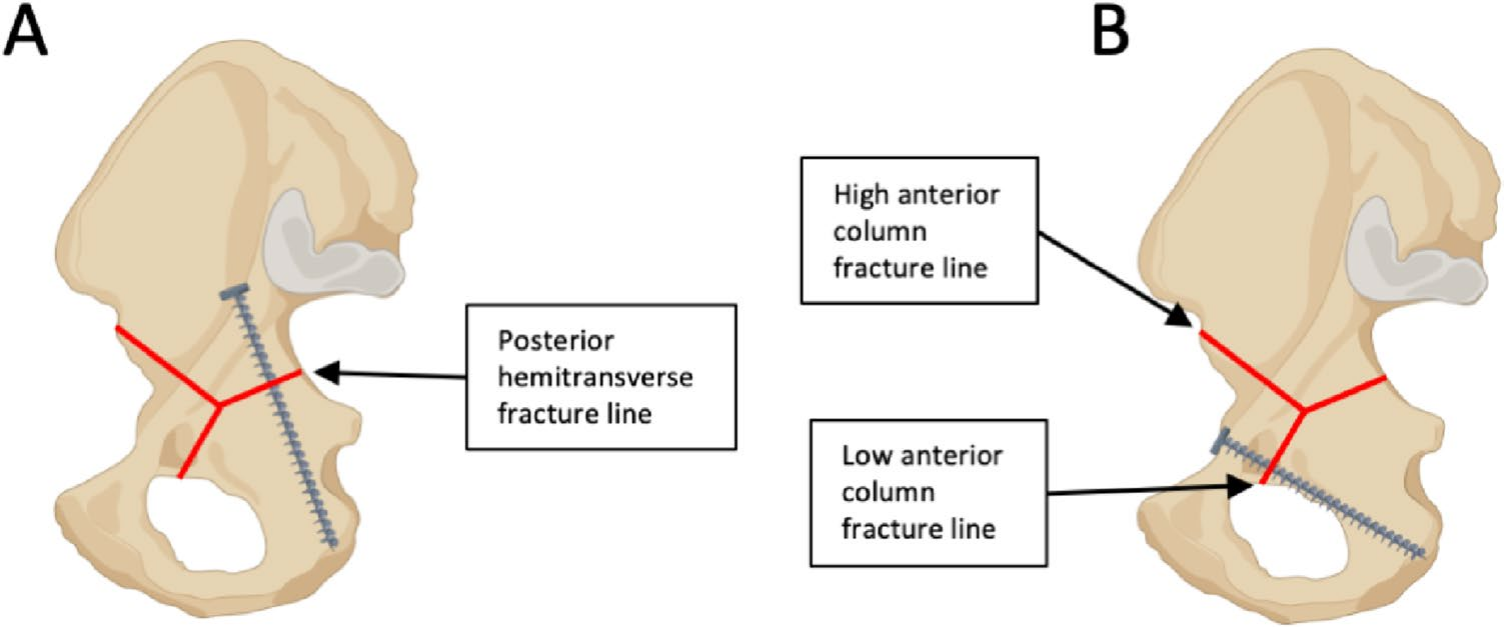
Screw courses of the antegrade posterior column screw (**A**) and of the infraacetabular screw (**B**) in relation to the three main fracture lines (red lines) of an ACPHT fracture. Created with BioRender.com

More precisely, it was demonstrated, that interfragmentary motion at the posterior hemitransverse fracture line (LX, shear XY) and at the high anterior column fracture line (LY) was significantly increased when an infraacetabular screw was used instead of an antegrade posterior column screw, each in combination with a suprapectineal plate. By contrast, fixation with an infraacetabular screw instead of an antegrade posterior column screw resulted in a reduced interfragmentary motion (LX, LY, LZ, shear XY) at the low anterior column fracture line. These results demonstrate that interfragmentary motion was increased at certain main fracture lines of the ACPHT fracture in both groups depending on the different screw courses. As it is illustrated in Fig. 5, the antegrade posterior column screw runs more perpendicular to the posterior hemitransverse fracture line and bridges the fracture line resulting in a higher fixation strength compared to the infraacetabular screw at this fracture line. In contrast, the infraacetabular screw runs more perpendicular to the low anterior column fracture line and bridges the fracture line resulting in a lower interfragmentary motion at this fracture line when an infraacetabular screw was used instead of an antegrade posterior column screw.

Besides an exact anatomical reduction, a biomechanically stable fracture fixation is one of the main predictors for the clinical and radiological outcome after surgically treated acetabulum fractures. Failure of osteosynthesis and secondary displacement due to an insufficient biomechanical stability increase the risk for a posttraumatic osteoarthritis of the hip joint [8, 11–13]. A remaining or secondary displacement was found to be clinically relevant only above 2–3 mm, since above this limit, the risk of a posttraumatic osteoarthritis is markedly increasing [8, 13]. The highest interfragmentary motions, which we measured in this biomechanical study, were 1.156 mm ± 0.337 mm (posterior hemitransverse fracture line, X-axis, IAS group) and 0.455 mm ± 0.130 mm (low anterior column fracture line, X-axis, APCS group) and thus were below the limit of 2–3 mm. Nevertheless, Hartel et al. showed that, especially in geriatric patients with osteoporotic bone, a biomechanically stable fixation of acetabulum fractures is pivotal for the radiological outcome [7]. In addition, a biomechanically stable fixation of acetabulum fractures is important to enable early mobilization of geriatric patients to avoid immobilization-associated complications [14–16]. It should be borne in mind that other factors also influence the risk for a posttraumatic osteoarthritis. For example, a dome impaction and a quadrilateral surface fragment, which were not considered in the ACPHT fracture model in this study, increase the risk for a posttraumatic osteoarthritis. Consequently, these risk factors should be included in the surgical planning and, particularly in geriatric patients, should be considered as a potential indication for primary arthroplasty [8, 46–48].

The increased interfragmentary motions observed in this study were not along the Z-axis, which would have led to compression of the fracture, but were shearing movements along the X-axis and Y-axis. It was shown that while axial compression on the fracture promotes bone healing, shear movements along the fracture line delay bone healing resulting in a decrease in mechanical stability [49, 50]. Thus, the increased interfragmentary motion at the main fracture lines along the X-axis and Y-axis could lead to a delayed union of the ACPHT fracture.

In addition to the conclusion from these data that an antegrade posterior column screw should not be replaced by an infraacetabular screw, it can be assumed that an infraacetabular screw as well as an antegrade posterior column screw may be used in combination to treat an ACPHT fracture, especially in cases where maximum stability is required. However, this hypothesis needs to be tested in further biomechanical studies. A FEA study of Wang et al. also demonstrated that an infraacetabular screw alone does not provide sufficient biomechanical stability for an isolated posterior column fracture but the biomechanical stability of a posterior column screw is increased with an additional infraacetabular screw [51]. This is in line with the concept of the infraacetabular screw, which was described by Culemann et al. in 2011: Closing of the periacetabular frame with an infraacetabular screw augments the biomechanical stability of acetabulum fractures involving both columns [35–39]. Graul et al. showed in a synthetic hemipelvis model with an ACPHT fracture that while the biomechanical stability of a two-dimensionally shaped conventional plate (J-plate) can be augmented with an infraacetabular screw, the addition of an infraacetabular screw does not result in an enhanced biomechanical stability of a three-dimensionally shaped plate, such as a suprapectineal plate with quadrilateral buttress [37]. In contrast, we demonstrated in this biomechanical analysis, that even when a suprapectineal plate is used, an infraacetabular screw significantly reduces interfragmentary motion at the low anterior column fracture line.

Some limitations of this biomechanical study should be considered. Although the test setup was developed to simulate the physiological loading forces and angles, which were described by Bergmann et al. in vivo, as accurately as possible, it still requires some simplifications and therefore only provides an approximation. Nevertheless, preliminary tests confirmed that the loading forces and angles are comparable to those observed in vivo, especially when compared to other available test setups for acetabulum fractures, and main muscle forces are included in this test setup [42–45]. A cyclic loading protocol with a total of 32,000 cycles was used, which is closer to a classical fatigue loading protocol with simulation of the postoperative phase than the majority of other available biomechanical test setups for acetabulum fractures [45]. However, a classical fatigue loading protocol requires 100,000 cycles or more and Olson et al. concluded that 200,000–250,000 cycles are necessary to cover the postoperative time until fracture healing [45, 52]. When interpreting the interfragmentary motion reported in this study, particularly with regard to the influence of shear motion on fracture healing, it must be noted that it represents a combined elastic–plastic deformation, since it was calculated as the difference between the 32,000th cycle with a primary load of 1800 N and the initial primary loading with 50 N at the start of cyclic loading protocol. However, in our opinion, this best represents the complex processes in vivo in the first months after fracture fixation. Although ACPHT fractures, especially in geriatric patients, are often accompanied by a dome impaction and a quadrilateral surface fragment, we used a standardized and previously validated ACPHT fracture model without dome impaction or a quadrilateral surface fragment in this biomechanical study [2, 8, 41, 53]. The reason for this was to focus on the stability of the two main columns of the acetabulum and especially on the fixation of the posterior column when assessing the effect of replacing the antegrade posterior column screw with an infraacetabular screw as well as to increase comparability with previous biomechanical studies by using this standardized and validated fracture model. A synthetic bone model as used in this biomechanical study provides standardized biomechanical conditions with a high comparability and reproducibility. On the other hand, simulation of the structural and mechanical properties of bone in vivo, especially of osteoporotic bone in elderly patients, is limited with synthetic bone substitutes.

## Conclusion

It was demonstrated that fixation of an ACPHT fracture with a suprapectineal plate combined with an infraacetabular screw instead of an antegrade posterior column screw resulted in an increased interfragmentary motion at the posterior hemitransverse fracture line and the high anterior column fracture line. Fixation with a suprapectineal plate combined with an antegrade posterior column screw but without an infraacetabular screw showed a higher interfragmentary motion at the low anterior column fracture line. Consequently, replacing the antegrade posterior column screw by an infraacetabular screw in the surgical treatment of acetabulum fractures with posterior column involvement can not be recommended. Future biomechanical studies may investigate whether the combination of an antegrade posterior column screw and an infraacetabular screw would be beneficial to obtain maximum stability.

## Data Availability

The datasets used and analyzed during the current study are available from the corresponding author on reasonable request.
